# Total flavonoid concentrations of bryophytes from Tianmu Mountain, Zhejiang Province (China): Phylogeny and ecological factors

**DOI:** 10.1371/journal.pone.0173003

**Published:** 2017-03-06

**Authors:** Xin Wang, Jianguo Cao, Xiling Dai, Jianbo Xiao, Yuhuan Wu, Quanxi Wang

**Affiliations:** 1 College of Life and Environmental Sciences, Shanghai Normal University, Shanghai, China; 2 Insititute of Applied Ecology, Chinese Academy of Sciences, Shenyang, China; 3 State Key Laboratory of Quality Research in Chinese Medicine, Institute of Chinese Medical Sciences, University of Macau, Taipa, Macau, China; 4 College of Life and Environmental Sciences, Hangzhou Normal University, Hangzhou, China; Northeast Forestry University, CHINA

## Abstract

The flavonoids in bryophytes may have great significance in phylogeny and metabolism research. However, to date there has been little research on bryophyte metabolites, especially flavonoids. To redress this somewhat, we determined flavonoid concentrations of bryophytes from Tianmu Mountain through a colorimetric assay and considered the factors influencing the results. This is the first time that the flavonoid contents of bryophytes have been examined in detail. The results revealed a range of total flavonoid concentrations in 90 samples collected from Tianmu Mountain from 1.8 to 22.3 mg/g (w/w). The total flavonoid contents of liverworts were generally higher than those of mosses; acrocarpous mosses had generally higher values than that of pleurocarpous mosses. The total flavonoid contents of bryophytes growing at lower light levels were general higher than those growing in full-sun. The total flavonoid contents of epiphytic bryophytes were highest, while those of aquatic bryophytes were the lowest. Total flavonoid contents of species growing at low-latitudes were much higher than those at high-latitude individuals. In conclusion, total flavonoid contents of bryophytes have some connection with plant phylogeny; more flavonoids might be contained in relatively primitive bryophytes. Meanwhile, the effects of ecological factors on total flavonoid contents of bryophytes exist; light and habitat (especially tree habitat and river habitat) might be representative factor.

## Introduction

Flavonoids, representing one type of plant secondary metabolites with over 10,000 known structures [1–2], are not only vital for plant growth and development [3], but also play an important role in the prevention and management of modern diseases [4–8]. Flavonoids are not restricted to vascular plants, but can also be found in bryophytes [9]. However, most flavonoid research has focused on the former, whilst bryophytes have only been sporadically studied [10–11].

Bryophytes, the oldest group of terrestrial plants [12], have experienced over 400 million years of extreme climatic change. Bryophytes are second only to angiosperms, within the kingdom Plantae, in the number of species in the group. Bryophyte flavonoids are of great significance in research on phylogeny and metabolism [13–14]. Extracts from bryophytes which contain flavonoids have been investigated extensively for their potential pharmacological applications: cytotoxic, anticancer and antitumor [15–17], antifungal [18–19], antibacterial [20–21] and antioxidant activities, and their ability to inhibit AChE activity [22].

Due to their small size, it is difficult to collect bryophyte samples in the field that are large enough for chemical experiments. Research on the chemical composition of bryophytes, especially flavonoids, is relatively rare. Globally, there are approximately 23 000 species of bryophytes, with 3021 species in China, of which about 50 species have been used in traditional Chinese medicine [23]. To date, flavonoids had been reported from only 1.4% of bryophyte species in China.

Tianmu Mountain (Tianmu Mountain National Nature Reserve, 30°18′30″-30°21′37″N, 119°24′11″-119°27′11″E) is located in Lin’an city, Zhejiang Province, China. The climate represents a transition from the mid-subtropics to the northern subtropics [24]. Sixty-seven species of liverwort, belonging to 32 genera and 24 families, and 220 species of moss, belonging to 152 genera and 65 families have been reported on Tianmu Mountain, growing under various environmental conditions [25]. Prior to this work, there had been no studies of the flavonoids in the bryophytes growing at the site.

This study specifically aims to address the following questions (1) what about the flavonoids of bryophytes from Tianmu Mountain? (2) Which factors could impact total flavonoid concentrations?

## Materials and methods

### Plant materials

The names of the authority who issued the permission for the two locations were Tianmu Mountain National Natural Reserve and E’erguna National Natural Reserve. Ninety samples, from 61 species and 31 families in S1 Table were collected from the Tianmu Mountain National Natural Reserve, Zhejiang Province (China) during April, June and July of 2013. Twenty-three samples, from 20 species and 9 families in S2 Table were collected from the E’erguna National Natural Reserve, Inner Mongolia (China) during July of 2013. The specimens were collected and identified by Yuhuan Wu who was familiar with bryoflora in Zhejiang Province. Voucher specimens were deposited in the College of Life & Environmental Science, Hangzhou Normal University (HTC).

### Chemicals and reagents

Details were presented in our previous report [20].

### Preparation of plant extracts

Fresh and undamaged plants were collected and stored for analysis. After being washed and dried in the shade, samples were further dried at 75°C for 2 days and then ground up. A portion of each dried sample (1.00 g) was extracted with 60% ethanol (25 mL) at 50°C for 2 h, followed by ultrasound-assisted extraction for 20 min. This process was performed twice. The extracts were filtered, and the volume of the solution kept constant at 50 mL.

### Determination of total flavonoid content

The total flavonoid concentrations were measured as described in our previous report [20]. A colorimetric assay was used to determine the total flavonoid content. The calibration curves for rutin would give A, B and r^2^ at 510 nm (OD). With the same way as rutin, total flavonoid contents of samples were determined. The formula was used as follows: total flavonoid content (mg/g) = [(OD_1_+OD_2_+OD_3_)/3–A]/B *10/2*volume/100*100%

### Statistical analysis

Statistical analysis was undertaken using R software, SPSS and Origin 7.5. Datas were reported as the mean of three independent samples.

## Results and discussion

### Total flavonoid content in 60% ethanol extracts from bryophytes of Tianmu Mountain

**Fig 1:**
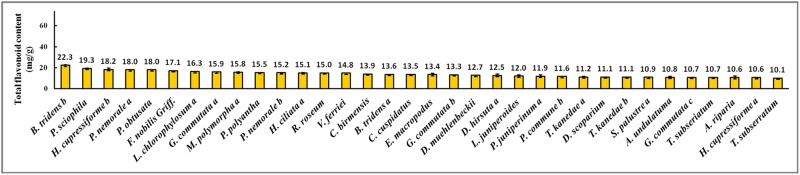
Bryophyte samples with total flavonoid concentration higher than 10.0 mg/g.

**Fig 2:**
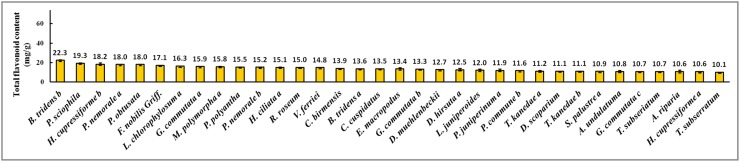
Bryophyte samples with total flavonoid concentration between 5.0 and 10.0 mg/g.

**Fig 3:**
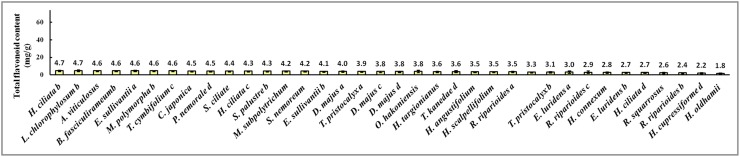
Bryophyte samples with total flavonoid concentration less than 5.0 mg/g.

**Fig 4:**
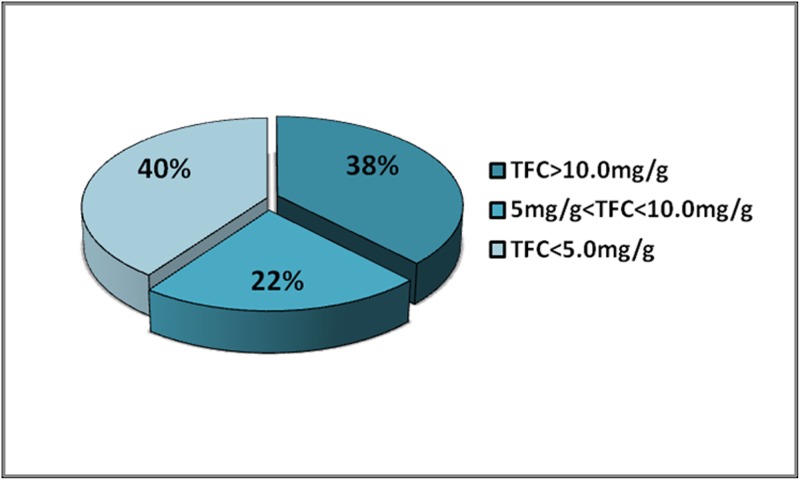
Total flavonoid concentration of bryophytes.

The total flavonoid concentrations found in the collected bryophytes were expressed as rutin equivalents in mg/g (w/w) and summarized in S1 Table. The range of total flavonoid concentrations in the 90 samples collected from Tianmu Mountain was from 1.8 to 22.3 mg/g (w/w). *Bazzania tridens* (Reinw., Blume & Nees). exhibited the highest total flavonoid content (22.3 mg/g), and *Hypnum oldhamii* (Mitt.) Jaeg. the lowest (1.8 mg/g). Thus, the total flavonoid content of *B*. *tridens* was about 12 times that of *H*. *oldhamii*. Figs 1–3 showed the concentrations for: the 34 samples for which the total flavonoid concentration was more than 10.0 mg/g; the 20 samples with concentrations between 5.0 and 10.0 mg/g; and the 36 samples with concentrations less than 5.0 mg/g. The total flavonoid contents of 62% of bryophytes from Tianmu Mountain were less than 10.0 mg/g (Fig 4).

Reviewing the ample literature on total flavonoid concentrations in plants, the range for spermatophyte species was from around 0.095 mg/g to 25.01 mg/g [26–32], and the total flavonoid content of most pteridophytes was reportedly greater than 50.0 mg/g [33–34]. Our results were clearly different from other researched plant groups. The total flavonoid contents of bryophytes were similar to those of spermatophytes, but far less than those of pteridophytes. This may be due to the differences in evolutionary status [11].

### The relationship between total flavonoid concentrations and phylogeny

Differences in the evolutionary status of bryophytes have been reported [35]. Some research suggested that liverworts were the earliest terrestrial plants [36], but other studies indicated that liverworts and mosses were combined as sister taxa [37]. Moreover, long-standing hypotheses relating to liverwort evolution had been questioned [38]. The mosses, the largest of all bryophyte groups, included both acrocarpous and pleurocarpous mosses [39], but the evolutional relationship between the two had long been controversial [40].

**Fig 5:**
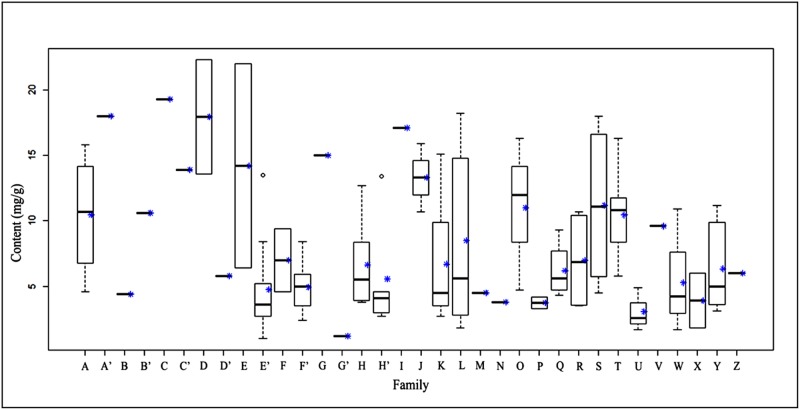
Relationship between flavonoid content and families of bryophytes. **Liverworts:** A = Marchantiaceae; A’ = Porellaceae; B = Scapaniaceae; B’ = Pallaviciniaceae; C = Plagiochilaceae; C’ = Lophoziaceae; D = Lepidoziaceae; D’ = Aytoniaceae; E = Ptilidiaceae. **Moss:** E’ = Amblystegiaceae; F = Anomodontaceae; F’ = Brachytheciaceae; G = Bryaceae; G’ = Climaciaceae; H = Dicranaceae; H’ = Entodontaceae; I = Fissidentaceae; J = Grimmiaceae; K = Hedwigiaceae; L = Hypnaceae; M = Hypopterygiaceae; N = Leskeaceae; O = Leucobryaceae; P = Meteoriaceae; Q = Mniaceae; R = Neckeraceae; S = Plagiotheciaceae; T = Polytrichaceae; U = Rhytidiaceae; V = Sematophyllaceae; W = Sphagnaceae; X = taxianke; Y = Thuidiaceae; Z = Trachypodaceae; *mean.

**Fig 6:**
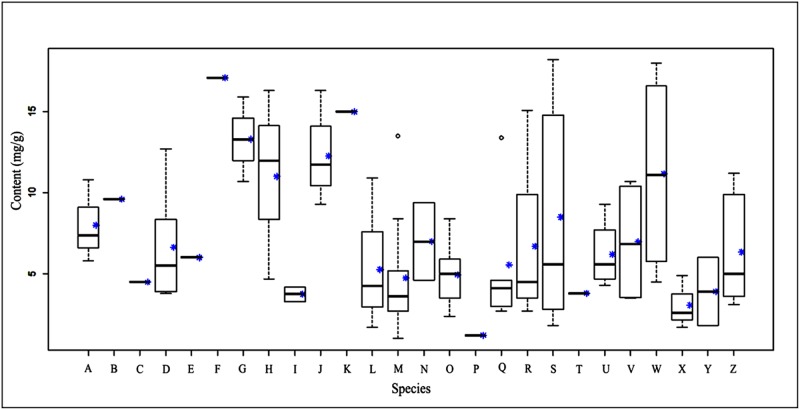
Relationship between flavonoid content and families of moss. **Acrocarpous:** A = Polytrichaceae; B = Sematophyllaceae; C = Hypopterygiaceae; D = Dicranaceae; E = Trachypodaceae; F = Fissidentaceae; G = Grimmiaceae; H = Leucobryaceae; I = Meteoriaceae; J = Polytrichaceae; K = Bryaceae; L = Sphagnaceae; **Pleurocarpous:** M = Amblystegiaceae; N = Anomodontaceae; O = Brachytheciaceae; P = Climaciaceae; Q = Entodontaceae; R = Hedwigiaceae; S = Hypnaceae; T = Leskeaceae; U = Mniaceae; V = Neckeraceae; W = Plagiotheciaceae; X = Rhytidiaceae; Y = Taxianke; Z = Thuidiaceae; *mean.

Herein, all samples, including the samples collected from E’erguna National Natural Reserve S2 Table, were separated into two groups, namely liverworts and mosses. The samples of mosses were also divided into two subgroups, acrocarpous mosses and pleurocarpous mosses. The comparison between liverworts and mosses with respect to total flavonoid content was shown in Fig 5, and the comparison between acrocarpous mosses and pleurocarpous mosses in Fig 6. To our knowledge, this was the first comparison of total flavonoid concentrations in bryophytes of different evolutionary status.

Since 1962 researchers have been attempting to use flavonoids to determine plant phylogenetic sequences. Although there was not a good fossil record, more and more evidences showed that flavonoid content was related to phylogeny [13–14]. Our data confirmed these results. Furthermore, we found that the total flavonoid content of liverworts was generally higher than that of mosses, and the mean total flavonoid content of Marchantiopsida (9.52 mg/g) was less than that of Jungermanniopsida (14.5 mg/g) in the liverworts. Acrocarpous mosses generally had higher concentrations than pleurocarpous ones, although interspecific differences were found.

The above results may suggest that more flavonoids might be contained in relatively primitive bryophytes. In recent report, more flavonoids were also found in primitive ferns [41]. Bryophyte and pteridophytes both belong to cryptogam, so it was speculated that primitive species might contain more flavonoids in cryptogam. In addition, the relationship between total flavonoid content and environmental factors should not be ignored.

### The relationship between total flavonoid concentrations and ecological factors

Flavonoids are important secondary metabolites in plants and are regulated by the environment. Dixon et al. (1995) found that changes in ecological factors associated with both abiotic and biotic stresses could alter the flavonoid content of plants [42]. Various ecological factors have been shown to have an impact on the secondary metabolite profile in angiospermae [43–44]. Nevertheless, the effects of environmental factors on the flavonoid content of bryophytes remain unclear.

**Fig 7:**
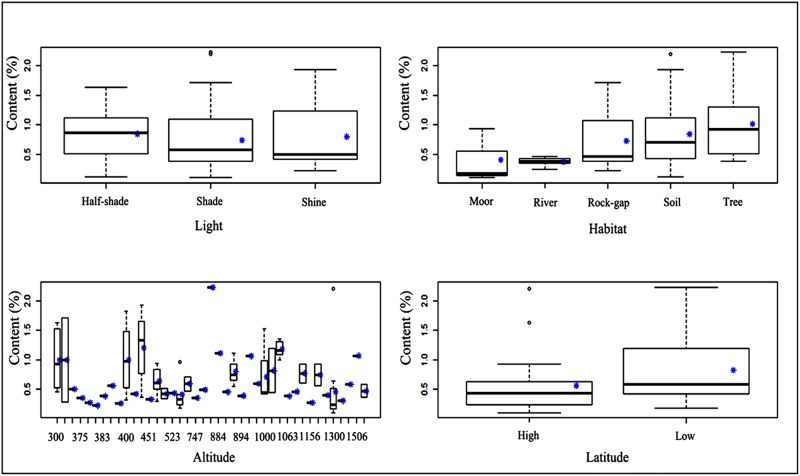
Relationships between ecological factors and total flavonoid content. **High latitude:** E’erguna National Natural Reserve; **Low latitude:** Tianmu Mountain National Natural Reserve.

The ecological factors that we examined were light, habitat, altitude, and latitude. Fig 7 showed the relationship between total flavonoid concentrations in bryophytes (113 samples) and these factors.

The effects of light on flavonoids in angiospermae have been shown to be significant [45–46]. Our results confirm this view. Total flavonoid concentrations in bryophytes growing in less sunshine conditions were generally higher than those in bryophytes growing in full sunshine. The results were same with pteridophytes [41]. The stress environment such as dimmer sunshine would promote the synthesis of flavonoids [47]. Light quality has been found that could alter flavonoid production [48–49].

It had been reported that there was no effect of altitude on total flavonoid concentrations in *Sphagnum junghuhnianum* in tropical montane forests of Borneo [50]. Our results were in accordance with this result. Total flavonoid concentrations in bryophytes exhibited no obvious relationship with altitude, but the interspecific differences were more obvious. Temperature decreases with the increase of altitude, which could raise the flavonoid content of bryophytes [51], this may be due to the distribution diversity of bryophytes.

The habitats of bryophytes were divided into moor, river, rock crevice, soil and tree. The results showed that total flavonoid concentrations in epiphytic bryophytes were the highest, while those of aquatic bryophytes the lowest. The results were also same with pteridophytes [41]. In addition, interspecific differences of epiphytic bryophytes were significant. This might be due to the fact that epiphytic species were exposed in the air and experience complex and varied environments, requiring them to synthesize flavonoids for biochemical protection. Conversely, aquatic bryophytes growing in a relatively constant environment were less influenced by ecological factors, so the protection conferred by flavonoids was less necessary. In addition, bryophytes living in rock-gap and soil were vulnerable to sunshine as well as other ecological factors, which leaded to lower flavonoid content than epiphytic bryophytes.

Total flavonoid content was higher in species growing at low latitudes than those at high latitudes, and interspecific differences of species at low latitudes were clear. This may be explained by the presence of different species at different latitudes.

Our results demonstrated that ecological factors do, indeed, influenced total flavonoid concentrations of bryophytes, as it the case in pteridophyte.

In conclusion, the range of total flavonoid concentrations of bryophytes from Tianmu Mountain was 1.8 to 22.3 mg/g (w/w), which was much lower than ferns. Total flavonoid contents of bryophytes have some connection with plant phylogeny, and more flavonoids might be contained in relatively primitive bryophytes. Meanwhile, the effects of ecological factors on total flavonoid contents of bryophytes existed; light and habitat (especially tree habitat and river habitat) might be main and representative factor.

## Supporting information

S1 Table Location, taxonomic information and total flavonoid concentrations of bryophytes from the Tianmu Mountain National Natural Reserve. (DOCX)

S2 Table Location, taxonomic information and total flavonoid concentrations in bryophytes from the E’erguna National Natural Reserve. (DOCX)
