# Correction: Total flavonoid concentrations of bryophytes from Tianmu Mountain, Zhejiang Province (China): Phylogeny and ecological factors

**DOI:** 10.1371/journal.pone.0179837

**Published:** 2017-06-12

**Authors:** Xin Wang, Jianguo Cao, Xiling Dai, Jianbo Xiao, Yuhuan Wu, Quanxi Wang

Fig 2 is incorrect. Please see the corrected Fig 2 here.

**Fig 2:**
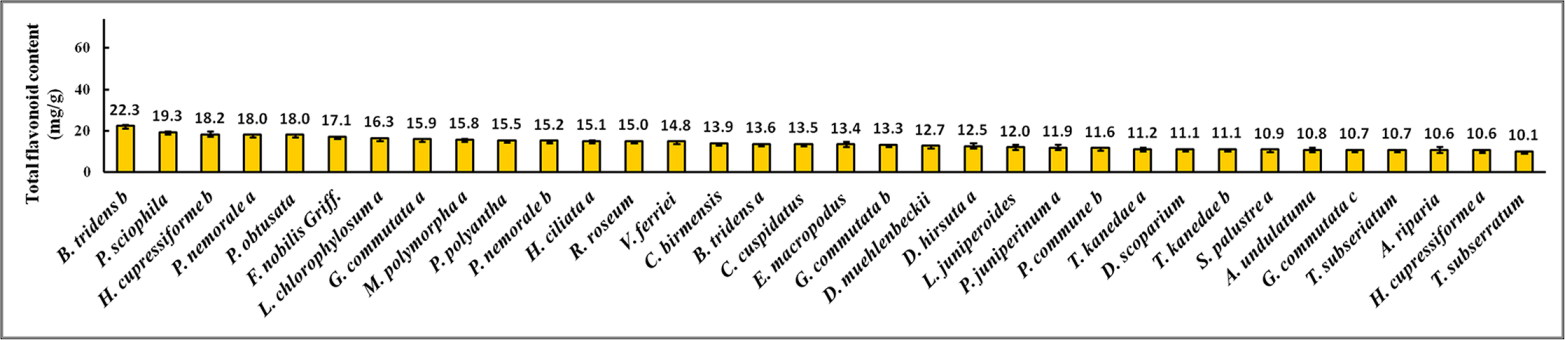
Bryophyte samples with total flavonoid concentration between 5.0 and 10.0 mg/g.
