# Correction: Total flavonoid concentrations of bryophytes from Tianmu Mountain, Zhejiang Province (China): Phylogeny and ecological factors

**DOI:** 10.1371/journal.pone.0192669

**Published:** 2018-02-06

**Authors:** The PLOS ONE Staff

Fig 2 is incorrect in the correction published on June 12, 2017. Please see the corrected Fig 2 here. The publisher apologizes for the error.

**Fig 2:**
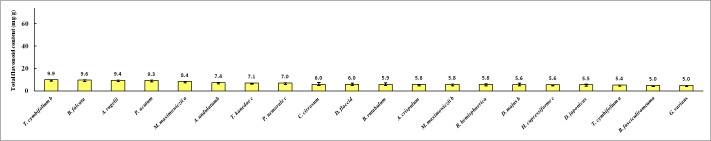
Bryophyte Samples with total flavonoid concentration between 5.0 and 10.0 mg/g.

## References

[1] Wang X, Cao J, Dai X, Xiao J, Wu Y, Wang Q (2017) Total flavonoid concentrations of bryophytes from Tianmu Mountain, Zhejiang Province (China): Phylogeny and ecological factors. PLoS ONE 12(3): e0173003 https://doi.org/10.1371/journal.pone.0173003 28263997 10.1371/journal.pone.0173003 PMC5338819

[2] Wang X, Cao J, Dai X, Xiao J, Wu Y, Wang Q (2017) Correction: Total flavonoid concentrations of bryophytes from Tianmu Mountain, Zhejiang Province (China): Phylogeny and ecological factors. PLoS ONE 12(6): e0179837 https://doi.org/10.1371/journal.pone.0179837 28604835 10.1371/journal.pone.0179837 PMC5467905

