# Supplementary material for: Total flavonoid concentrations of bryophytes from Tianmu Mountain, Zhejiang Province (China): Phylogeny and ecological factors
Source: PLoS One. 2017 Mar 6;12(3):e0173003. doi: 10.1371/journal.pone.0173003 (PMC5338819; doi:10.1371/journal.pone.0173003)
Supplement: S1 Table — (DOCX) [file pone.0173003.s001.docx]

**S1 Table. Location, taxonomic information and total flavonoid concentrations of bryophytes from the Tianmu Mountain National Natural Reserve.**

| **NO.** | **Family** | **Species** | **Habitat** | **Altitude** | **Concentration**  **mg/g** |
| --- | --- | --- | --- | --- | --- |
| 2013042209 | Anomodontaceae | *Anomodon rugelii* (Müll. Hal. ) Keissl. | Shade  Rock crevice | 500 | 9.4±0.25 |
| 2013042115 | Anomodontaceae | *Anomodon viticulosus* (Hedw.) Hask et Tayl. | Full sun  Rock crevice | 300 | 4.6±0.11 |
| 2013042257 | Pallaviciniaceae | *Aplozia riparia* (Tayl.) Dumort. | Half-shade  Rock crevice | 400 | 10.6±1.57 |
| 2013061030 | Polytrichaceae | *Atrichum crispulum* Schimp. *ex* Besch. | Shade  Soil | 1481 | 5.8±0.21 |
| 2013041811 | Polytrichaceae | *Atrichum undulatum* (Hedw.) P. Beauv. -a | Shade  Soil | 890 | 7.4±0.21 |
| 20130418101 | Polytrichaceae | *Atrichum undulatum* (Hedw.) Lindb.b | Shade  Soil | 1050 | 10.8±1.13 |
| 2013042208 | Lepidoziaceae | *Bazzania tridens* (Reinw., Blume & Nees) Trevis. -a | Shade  Tree | 450 | 13.6±0.14 |
| 2013070413 | Lepidoziaceae | *Bazzania tridens* (Reinw., Blume & Nees) Trevis. -b | Shade  Tree | 831 | 22.3±0.50 |
| 20130422011 | Brachytheciaceae | *Brachythecium fasciculirameum* Müll. Hal. -a | Full sun  Soil | 500 | 5±1.15 |
| 2013042230 | Brachytheciaceae | *Brachythecium fasciculirameum* Müll. Hal. -b | Shade  Tree | 400 | 4.6±0.04 |
| 2013070426 | Brachytheciaceae | *Brachythecium rutabulum* (Hedw.) B.S.G. | Full sun  Rock crevice | 935 | 5.9±1.57 |
| 2013042242 | Sematophyllaceae | *Brotherella falcata* Broth. | Shade  Soil | 600 | 9.6±0.44 |
| 2013048110 | Amblystegiaceae | *Calliergonella cuspidatus* (Hedw.) Loeske | Shade  Soil | 1050 | 13.5±0.17 |
| 2013042254 | Lophoziaceae | *Chandonanthus birmensis* Steph. | Full sun  Rock crevice | 400 | 13.9±0.04 |
| 2013042234 | Brachytheciaceae | *Cirriphyllum cirrosum* (Schwaegr.) Grout | Shade  Rock crevice | 500 | 6±1.57 |
| 2013070433 | Hypopterygiaceae | *Cyathophorella japonica* Broth. | Full sun  Rock crevice | 1000 | 4.5±0.28 |
| 2013041810 | Dicranaceae | *Dicranum japonicas* Broth. | Half-shade  Soil | 890 | 5.5±0.54 |
| 2013061019 | Dicranaceae | *Dicranum majus* Turn. -a | Shade  Soil | 1291 | 4±0.67 |
| 2013061011 | Dicranaceae | *Dicranum majus* Turn. -b | Shade  Tree | 1229 | 5.6±1.57 |
| 2013070202 | Dicranaceae | *Dicranum majus* Turn. -c | Shade  Rock crevice | 383 | 3.8±0.35 |
| 2013070439 | Dicranaceae | *Dicranum majus* Turn. -d | Half- shade  Rock crevice | 1063 | 3.8±0.07 |
| 2013048104 | Dicranaceae | *Dicranum muehlenbeckii* Bruch & Schimp. | Shade  Soil | 1050 | 12.7±0.23 |
| 2013070417 | Dicranaceae | *Dicranum scoparium* Hedw. | Shade  Rock crevice | 884 | 11.1±0.32 |
| 2013042121 | Marchantiaceae | *Dumortiera hirsuta* (Sw.) Reinw., Bl. & Nees a | Shade  Tree | 300 | 12.5±1.57 |
| 2013042117 | Trachypodaceae | *Duthiella flaccid* (Card.) Broth. | Shade  Rock crevice | 300 | 6±1.00 |
| 2013061022 | Entodontaceae | *Entodon luridens* (Griff.) Jaeg. -a | Full sun  Soil | 1301 | 3±1.53 |
| 2013061053 | Entodontaceae | *Entodon luridens* (Griff.) Jaeg. -b | Shade  Rock crevice | 1156 | 2.7±0.07 |
| 20130418106 | Entodontaceae | *Entodon macropodus* (Hedw.) Müll. Hal. | Shade  Rock crevice | 1050 | 13.4±1.51 |
| 2013042201 | Entodontaceae | *Entodon sullivantii* (Müll. Hal.) Lindb. -a | Shade  Rock crevice | 300 | 4.6±0.60 |
| 2013042222 | Entodontaceae | *Entodon sullivantii* (Müll. Hal.) Lindb. -b | Full sun  Rock crevice | 600 | 4.1±0.07 |
| 2013070101 | Fissidentaceae | *Fissidens nobilis* Griff. | Shade  Rock crevice | 331 | 17.1±0.11 |
| 2013061121 | Hypnaceae | *Gollania varians (Mitt.) Broth.* | Full sun  Rock crevice | 351 | 5±0.20 |
| 2013042255 | Grimmiaceae | *Grimmia commutata Hueb. -a* | Half-shade  Rock crevice | 400 | 15.9±0.28 |
| 2013042205 | Grimmiaceae | *Grimmia commutata Hueb.b* | Shade  Rock crevice | 450 | 13.3±0.14 |
| 2013061045 | Grimmiaceae | *Grimmia commutata Hueb.c* | Full sun  Rock crevice | 1506 | 10.7±0.32 |
| 2013061135 | Thuidiaceae | *Haplocladium angustifolium (Hampe et Müll. Hal.) Broth.* | Full sun  Rock crevice | 375 | 3.5±0.26 |
| 2013042203 | Hedwigiaceae | *Hedwigia ciliata (Hedw.) Ehrh. ex P. Peauv. -a* | Shade  Rock crevice | 300 | 15.1±0.35 |
| 2013070402 | Hedwigiaceae | *Hedwigia ciliata (Hedw.) Ehrh. ex P. Peauv. -b* | Full sun  Rock crevice | 730 | 4.7±0.67 |
| 2013070306 | Hedwigiaceae | *Hedwigia ciliata (Hedw.) Ehrh. ex P. Peauv. -c* | Shade  Rock crevice | 450 | 4.3±0.32 |
| 2013061058 | Hedwigiaceae | *Hedwigia ciliata (Hedw.) Ehrh. ex P. Peauv. -d* | Shade  Rock crevice | 377 | 2.7±0.07 |
| 2013042204 | Neckeraceae | *Homaliadelphus targionianus (Mitt.) Dix. et P. Varde* | Full sun  Rock crevice | 450 | 3.6±0.57 |
| 2013070406 | Neckeraceae | *Homaliodendron scalpellifolium Fleisch.* | Shade  Rock crevice | 747 | 3.5±0.04 |
| 2013070105 | Hypnaceae | *Homomallium connexum (Card.) Broth.* | Shade  Rock crevice | 331 | 2.8±0.49 |
| 2013041812 | Hypnaceae | *Hypnum cupressiforme L. ex Hedw. -a* | Half-shade  Soil | 900 | 10.6±0.07 |
| 2013042253 | Hypnaceae | *Hypnum cupressiforme L. ex Hedw. -b* | Full sun  Soil | 400 | 18.2±1.57 |
| 2013070204 | Hypnaceae | *Hypnum cupressiforme L. ex Hedw. -c* | Half-shade  Soil | 385 | 5.6±0.07 |
| 2013070106 | Hypnaceae | *Hypnum cupressiforme L. ex Hedw. -d* | Full sun  Rock crevice | 378 | 2.2±0.46 |
| 2013042221 | Hypnaceae | *Hypnum oldhamii (Mitt.) Jaeg.* | Shade  Soil | 600 | 1.8±0.30 |
| 2013042112 | Leucobryaceae | *Leucobryum chlorophylosum Müll. Hal. -a* | Shade  Rock crevice | 300 | 16.3±0.25 |
| 2013070305 | Leucobryaceae | *Leucobryum chlorophylosum Müll. Hal. -b* | Shade  Rock crevice | 450 | 4.7±1.08 |
| 2013042227 | Leucobryaceae | *Leucobryum juniperoides (Brid.) Müll. Hal.* | Shade  Tree | 400 | 12±1.43 |
| 2013042114 | Marchantiaceae | *Marchantia polymorpha L. -a* | Half-shade  Rock crevice | 300 | 15.8±0.50 |
| 2013070447 | Marchantiaceae | *Marchantia polymorpha L. -b* | Shade  Rock crevice | 1079 | 4.6±0.14 |
| 2013070432 | Meteoriaceae | *Meteorium subpolytrichum (Besch.) Broth.* | Full sun  Rock crevice | 1000 | 4.2±0.44 |
| 2013070307 | Meteoriaceae | *Meterium helminthocladulum (Card.) Broth.* | Shade  Rock crevice | 451 | 3.3±0.07 |
| 2013042101 | Brachytheciaceae | *Myuroclada maximowiczii (Borszcz.) Steere et Schof. -a* | Half-shade  Soil | 300 | 8.4±0.11 |
| 2013061037 | Brachytheciaceae | *Myuroclada maximowiczii (Borszcz.) Steere et Schof. -b* | Full sun  Soil | 1507 | 5.8±0.49 |
| 2013042224 | Leskeaceae | *Okamuraea hakoniensis (Mitt.) Broth.* | Shade  Tree | 400 | 3.8±1.57 |
| 20130422010 | Plagiochilaceae | *Plagiochila sciophila Nees ex Lindenb.* | Full sun  Soil | 450 | 19.3±0.34 |
| 2013061006 | Mniaceae | *Plagiomnium acutum (Lindb.) T. Kop.* | Shade  Soil | 1229 | 9.3±0.50 |
| 2013042207 | Plagiotheciaceae | *Plagiothecium nemorale (Mitt.) Jaeg. -a* | Full sun  Soil | 450 | 18±0.25 |
| 2013042264 | Plagiotheciaceae | *Plagiothecium nemorale (Mitt.) Jaeg. -b* | Shade  Soil | 1000 | 15.2±0.04 |
| 2013042226 | Plagiotheciaceae | *Plagiothecium nemorale (Mitt.) Jaeg. -c* | Shade  Soil | 400 | 7±0.23 |
| 2013070418 | Plagiotheciaceae | *Plagiothecium nemorale (Mitt.) Jaeg. -d* | Shade  Rock crevice | 886 | 4.5±0.21 |
| 20130418109 | Polytrichaceae | *Polytrichum commune Hedw.b* | Shade  Soil | 1050 | 11.6±0.21 |
| 2013070438 | Polytrichaceae | *Polytrichum juniperinum Hedw. -a* | Half-shade  Soil | 1008 | 11.9±1.43 |
| 2013042206 | Porellaceae | *Porella obtusata* var. *macroloba* (Steph.) Hatt. & Zhang | Full sun  Soil | 450 | 18±0.32 |
| 2013042123 | Hypnaceae | *Pylaisiella polyantha* (Hedw.) Grout | Full sun  Rock crevice | 300 | 15.5±0.07 |
| 2013042113 | Aytoniaceae | *Reboulia hemisphaerica* (L.) Raddi. | Shade  Soil | 300 | 5.8±0.67 |
| 2013061101 | Bryaceae | *Rhodobryum roseum* (Weis.) Limpr. | Shade  Soil | 450 | 15±0.32 |
| 2013042214 | Brachytheciaceae | *Rhynchostegium riparioides* (Hedw.) Card. -a | Shade  River | 600 | 3.5±0.73 |
| 2013042215 | Brachytheciaceae | *Rhynchostegium riparioides* (Hedw.) Card. -b | Shade  River | 600 | 2.4±0.11 |
| 2013042231 | Brachytheciaceae | *Rhynchostegium riparioides* (Hedw.) Card. -c | Shade  Soil | 500 | 2.9±1.57 |
| 2013070205 | Rhytidiaceae | *Rhytidiadelphus squarrosus* (Hedw.) Warnst. | Half-shade  Soil | 386 | 2.6±0.52 |
| 2013070437 | Porellaceae | *Scapania ciliate* S. Lac. *in* Miguel | Shade  Rock crevice | 1008 | 4.4±0.18 |
| 2013070303 | Sphagnaceae | *Sphagnum nemoreum* Scop. | Shade  Rock crevice | 443 | 4.2±0.28 |
| 20130418111 | Sphagnaceae | *Sphagum palustre* L. -a | Shade  Soil | 1050 | 10.9±0.32 |
| 2013070436 | Sphagnaceae | *Sphagum palustre* L. -b | Shade  Soil | 1000 | 4.3±0.10 |
| 20130422191 | Neckeraceae | *Thamnobryum subseriatum* (Mitt. *ex* S. Lac.) Tan | Half-shade  Rock crevice | 450 | 10.7±0.25 |
| 2013042155 | Neckeraceae | *Thamnobryum subserratum* (Hook.) Nog. & Iwats. | Shade  Rock crevice | 300 | 10.1±0.04 |
| 2013042232 | Thuidiaceae | *Thuidium cymbifolium* (Dozy et Molk.) Dozy et Molk. -a | Half-shade  Rock crevice | 400 | 5.4±1.56 |
| 20130418103 | Thuidiaceae | *Thuidium cymbifolium* (Dozy et Molk.) Dozy et Molk. -b | Shade  Soil | 1050 | 9.9±0.07 |
| 2013042109 | Thuidiaceae | *Thuidium cymbifolium* (Dozy et Molk.) Dozy et Molk. -c | Full sun  River | 300 | 4.6±0.28 |
| 20130422192 | Thuidiaceae | *Thuidium kanedae* Sak. -a | Half-shade  Soil | 450 | 11.2±0.71 |
| 2013041806 | Thuidiaceae | *Thuidium kanedae* Sak. -b | Half-shade  Soil | 890 | 11.1±0.18 |
| 2013070401 | Thuidiaceae | *Thuidium kanedae* Sak. -c | Full sun  Rock crevice | 730 | 7.1±0.04 |
| 2013061039 | Thuidiaceae | *Thuidium kanedae* Sak. -d | Full sun  Soil | 1507 | 3.6±1.57 |
| 2013070421 | Thuidiaceae | *Thuidium pristocalyx* (Müll. Hal.) Jaeg. -a | Shade  Rock crevice | 894 | 3.9±0.47 |
| 2013042212 | Thuidiaceae | *Thuidium pristocalyx* (Müll. Hal.) Jaeg. -b | Full sun  Rock crevice | 600 | 3.1±0.73 |
| 2013042251 | Hypnaceae | *Vesicularia ferriei* (Card. et Ther.) Broth. | Full sun  Rock crevice | 400 | 14.8±0.46 |
