# Supplementary material for: Total flavonoid concentrations of bryophytes from Tianmu Mountain, Zhejiang Province (China): Phylogeny and ecological factors
Source: PLoS One. 2017 Mar 6;12(3):e0173003. doi: 10.1371/journal.pone.0173003 (PMC5338819; doi:10.1371/journal.pone.0173003)
Supplement: S2 Table — (DOCX) [file pone.0173003.s002.docx]

**S2 Table. Location, taxonomic information and total flavonoid concentrations in bryophytes from the E’erguna National Natural Reserve.**

| **NO.** | **Family** | **Species** | **Habitat** | **Altitude** | **Concentration**  **mg/g** |
| --- | --- | --- | --- | --- | --- |
| 2013071217 | Amblystegiaceae | *Amblystegium* *serpens* (Hedw.) B.S.G. | Shade  Soil | 1300 | 2±0.32 |
| 2013071224 | Amblystegiaceae | *Calliergon* *giganteum* (Schimp. ) Kindb. | Full sun  River | 1300 | 4.3±0.01 |
| 2013071466 | Amblystegiaceae | *Cratoneuron* *filicinum* (Hedw. ) Spruce | Half-shade  Rock crevice | 400 | 3.2±0.21 |
| 2013071422 | Amblystegiaceae | *Warnstorfia* *fluitans* (Hedw.) Loeske | Half-shade  Soil | 400 | 5.2±0.05 |
| 2013071221 | Amblystegiaceae | *Sanionia* *uncinatus* (Hedw.) Loeske | Shade  Soil | 522 | 3.4±0.05 |
| 2013071121 | Amblystegiaceae | *Drepanocladus* *aduncus* var. *kneiffii* (Schimp. *in* B.S.G.) Mönk. b | Shade  Moor | 1300 | 1±0.02 |
| 2013071260 | Amblystegiaceae | *Drepanocladus* *aduncus* var. *kneiffii* (Schimp. *in* B.S.G.) Mönk. a | Shade  River | 1300 | 3.8±0.07 |
| 2013071727 | Amblystegiaceae | *Drepanocladus* *vernicosus* (Lindb.) Warnst. | Full sun  Soil | 500 | 8.4±0.03 |
| 2013071226 | Amblystegiaceae | *Hygrohypnum* *ochraceum* (Turn. *ex* Wils.) Loeske | Shade  Rock crevice | 1300 | 2.7±0.07 |
| 2013071232 | Climaciaceae | *Climacium* *dendroides* (Hedw.) F. Weber & D. Mohr | Half-shade  Soil | 1300 | 1.2±0.04 |
| 2013071420 | Marchantiaceae | *Dumortiera* *hirsuta* (Sw.) Reinw., Bl. & Nees -b | Half-shade  Soil | 400 | 8.9±0.02 |
| 2013071354 | Mniaceae | *Plagiomnium* *cuspidatum* (Hedw. ) T. Kop. | Shade  Soil | 523 | 4.3±0.03 |
| 2013071225 | Mniaceae | *Plagiomnium* *rostratum* (Schrad. ) T. Kop. | Shade  Soil | 1100 | 6.1±0.02 |
| 2013071220 | Mniaceae | *Plagiomnium* *venustum* (Mitt. ) T. Kop. | Shade  Soil | 522 | 5.1±0.01 |
| 2013071314 | Taxianke | *Pleurozium* *schreberi* (Brid.) Mitt. -a | Shade  Soil | 1300 | 6±0.10 |
| 2013071521 | Taxianke | *Pleurozium* *schreberi* (Brid.) Mitt. -b | Shade  Soil | 1300 | 1.8±0.03 |
| 2013071509 | Polytrichaceae | *Polytrichum* *commune* Hedw.a | Half-shade  Soil | 400 | 16.3±0.05 |
| 2013071128 | Polytrichaceae | *Polytrichum* *juniperinum* Hedw. -b | Shade  Moor | 1100 | 9.3±0.01 |
| 2013071301 | Ptilidiaceae | *Ptilidium* *pulcherrimum* (Web.) Hapm. -a | Shade  Soil | 1300 | 22±0.03 |
| 20130711281 | Ptilidiaceae | *Ptilidium* *pulcherrimum* (Web.) Hapm. -b | Shade  Tree | 1300 | 6.4±0.03 |
| 2013071462 | Rhytidiaceae | *Rhytidiadelphus* *triquetrus* (Hedw.) Warnst. | Half-shade  Soil | 800 | 4.9±0.01 |
| 2013071257 | Rhytidiaceae | *Rhytidium* *rugosum* (Hedw.) Kindb. | Shade  Soil | 1300 | 1.7±0.08 |
| 2013071120 | Sphagnaceae | *Sphagum* *palustre* L. -c | Shade  Moor | 1300 | 1.7±0.16 |
